# Correction: Häyrinen et al. The Transcription Factor Twist1 Has a Significant Role in Mycosis Fungoides (MF) Cell Biology: An RNA Sequencing Study of 40 MF Cases. *Cancers* 2023, *15*, 1527

**DOI:** 10.3390/cancers17010152

**Published:** 2025-01-06

**Authors:** Marjaana J. Häyrinen, Jenni Kiiskilä, Annamari Ranki, Liisa Väkevä, Henry J. Barton, Milla E. L. Kuusisto, Katja Porvari, Hanne Kuitunen, Kirsi-Maria Haapasaari, Hanna-Riikka Teppo, Outi Kuittinen

**Affiliations:** 1 Institute of Clinical Medicine, Faculty of Health Medicine, University of Eastern Finland, 70210 Kuopio, Finland; 2 Cancer Research and Translational Medicine Research Unit, University of Oulu, 90014 Oulu, Finland; 3 Department of Skin and Allergic Diseases, University of Helsinki, Helsinki University Central Hospital, P.O. Box 160, 00029 HUS Helsinki, Finland; 4 Genevia Technologies Oy, 33100 Tampere, Finland; 5 Department of Haematology, Oulu University Hospital, 90220 Oulu, Finland; 6 Medical Research Center Oulu, Oulu University Hospital, University of Oulu, 90220 Oulu, Finland; 7 Cancer Center, Oulu University Hospital, 90220 Oulu, Finland; 8 Department of Pathology, Oulu University Hospital, 90220 Oulu, Finland; 9 Cancer Center, Kuopio University Hospital, 70210 Kuopio, Finland

## Error in Figure

In the original publication [1], there was a mistake in Figure 6 as published. The Figure was missing data points. The corrected Figure 6 appears below. The authors apologize for any inconvenience caused and state that the scientific conclusions are unaffected. This correction was approved by the Academic Editor. The original publication has also been updated.

## Figures and Tables

**Figure 6:**
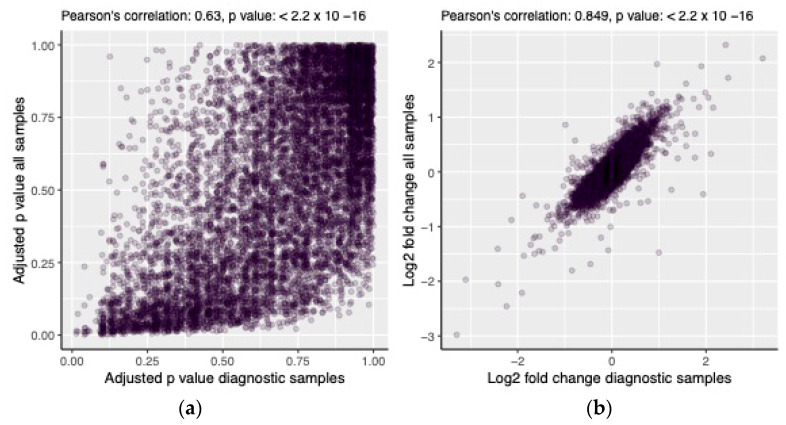
Comparison of the results of the DE analysis run on diagnostic samples and all samples combined, showing the relationship between the two models’ adjusted *p* values (**a**) and predicted log2 fold changes (**b**).
