# Supplementary material for: The Transcription Factor Twist1 Has a Significant Role in Mycosis Fungoides (MF) Cell Biology: An RNA Sequencing Study of 40 MF Cases
Source: Cancers (Basel). 2023 Feb 28;15(5):1527. doi: 10.3390/cancers15051527 (PMC10000433; doi:10.3390/cancers15051527)
Supplement: Supplementary file 1 [file cancers-15-01527-s001.zip › cancers-2199028 - Supplementary materials.pdf]

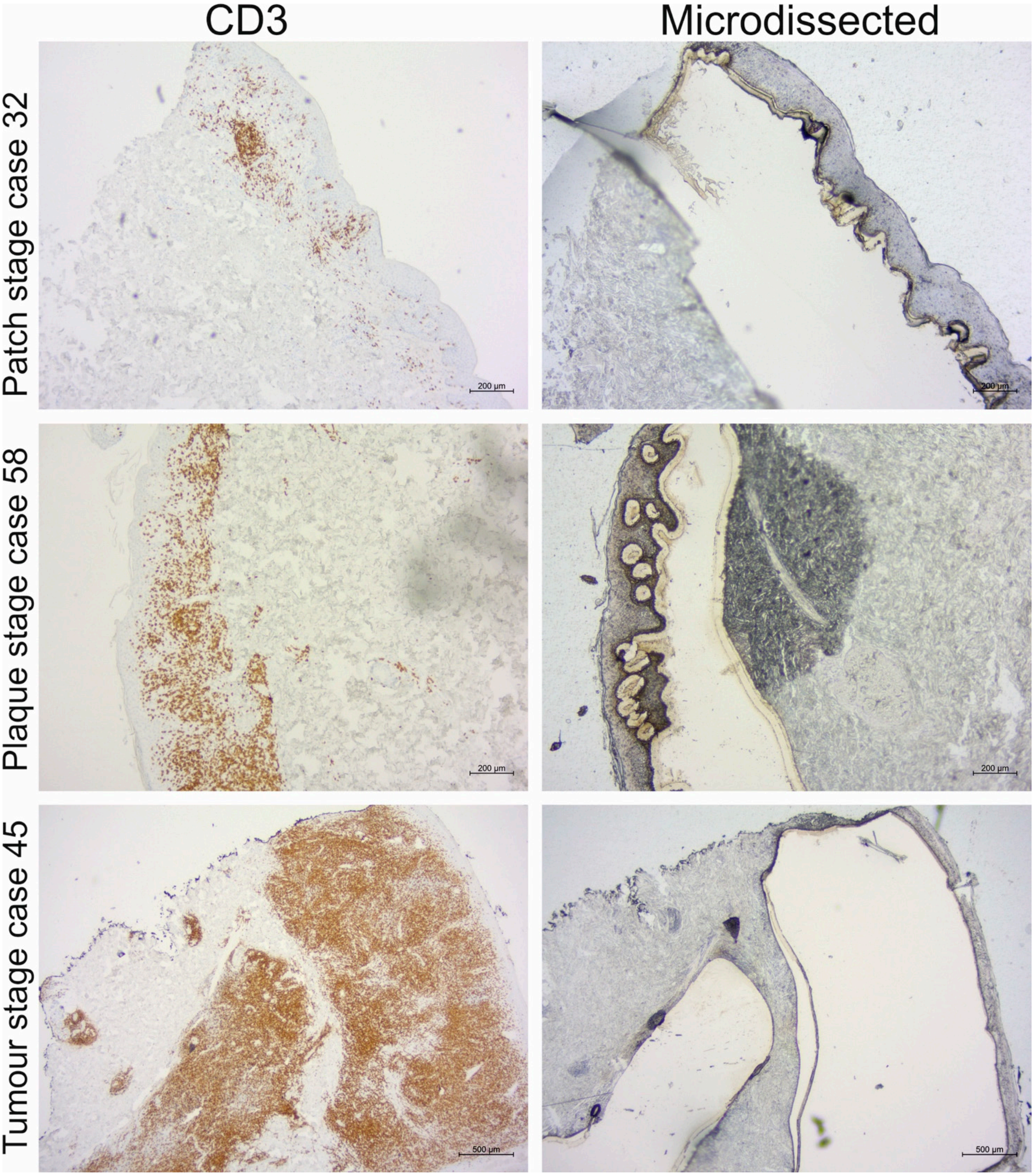

Figure S1. The immunohistochemical CD3 expression and microdissected region.

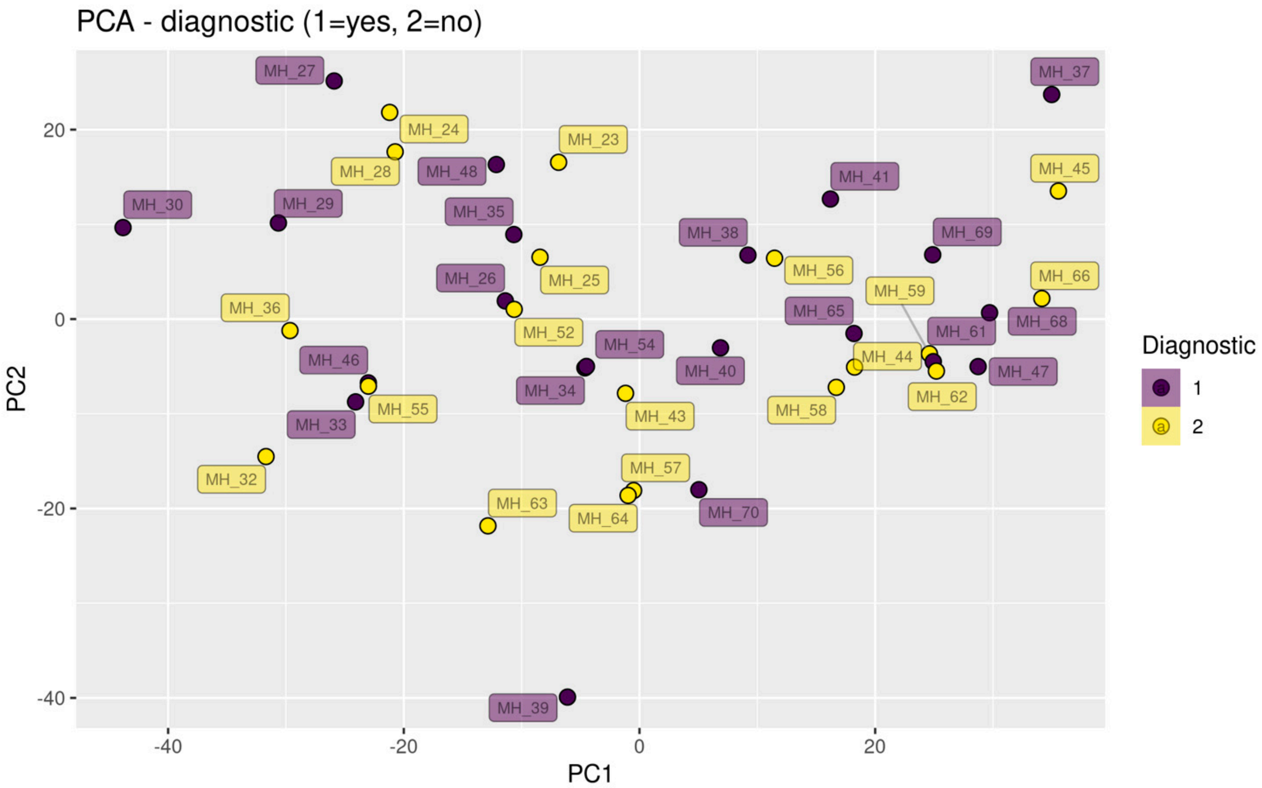

Figure S2. Principal component analysis with diagnostic status annotated.

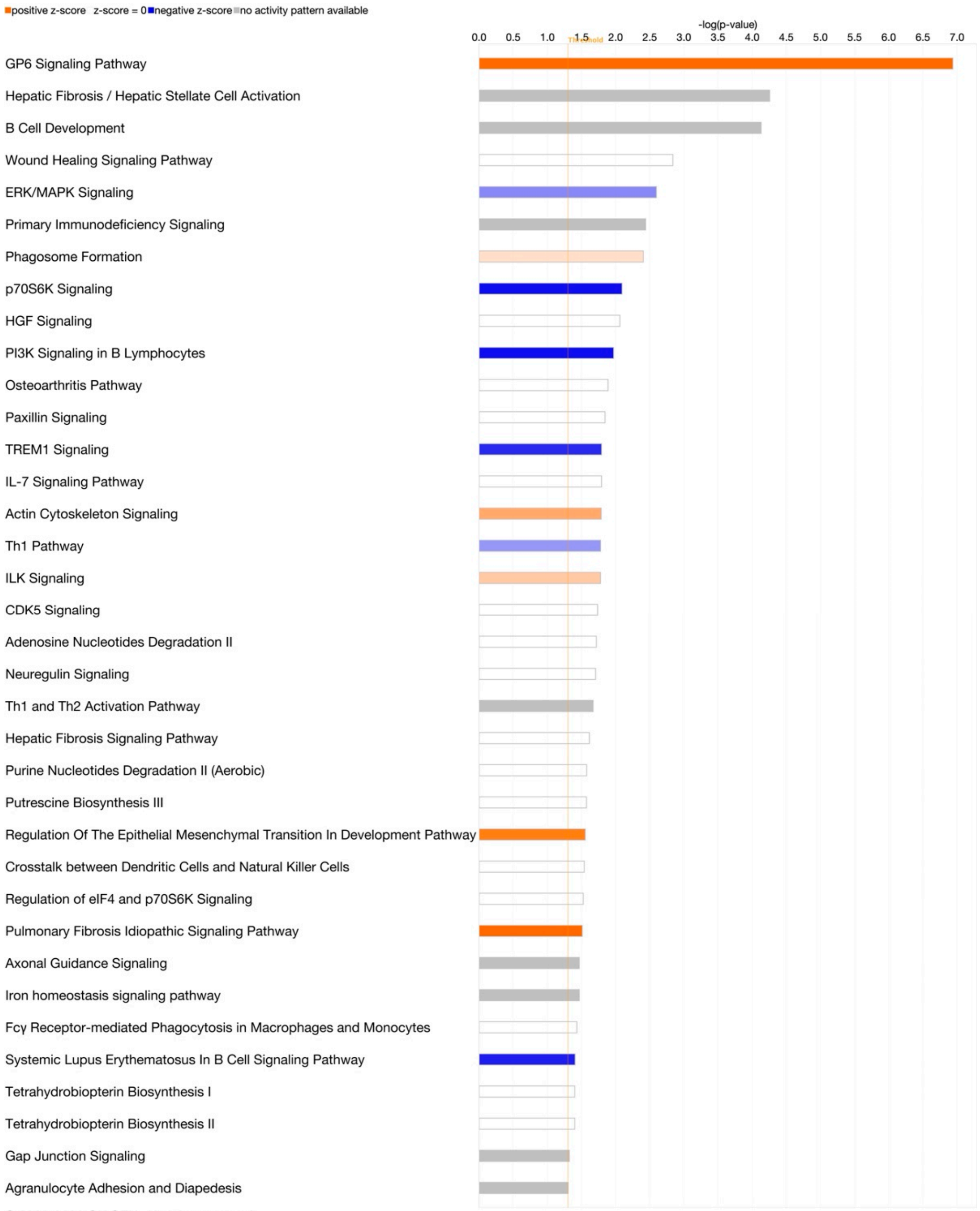

© 2000-2022 QIAGEN. All rights reserved.

Figure S3. Canonical pathways significant before correcting for multiple testing. Orange indicates the net up-regulation of a pathway, blue indicates down-regulation, and grey means that IPA could not predict the activity of the pathway. GP6, glycoprotein VI; ERK, extracellular signal-regulated kinase; MAPK, mitogen-activated protein kinase; p70S6K, ribosomal protein S6 kinase beta-1; HGF, hepatocyte growth factor; PI3K, phosphoinositide 3-kinase; TREM, triggering receptor expressed on myeloid cells 1; IL-7, interleukin 7, Th1; T helper 1, ILK, integrin-linked kinase; CDK5, cyclin-dependent kinase 5; Th 2, T helper 2; eIF4, eukaryotic translation initiation factor 4E.
